# Microstructure and Mechanical/Elastic Performance of Biobased Poly (Butylene Furanoate)–*Block*–Poly (Ethylene Oxide) Copolymers: Effect of the Flexible Segment Length

**DOI:** 10.3390/polym12020271

**Published:** 2020-01-28

**Authors:** Magdalena Kwiatkowska, Inez Kowalczyk, Konrad Kwiatkowski, Agata Zubkiewicz

**Affiliations:** 1 Department of Materials Technology, West Pomeranian University of Technology, Piastow Av. 19, 70-310 Szczecin, Poland; inez.kowalczyk@zut.edu.pl; 2 Department of Mechanics, West Pomeranian University of Technology, Piastow Av. 19, 70-310 Szczecin, Poland; konrad.kwiatkowski@zut.edu.pl; 3 Department of Technical Physics, West Pomeranian University of Technology, Piastow Av. 19, 70-310 Szczecin, Poland; agata.zubkiewicz@zut.edu.pl

**Keywords:** furan-ester copolymers, thermoplastic elastomers, phase separation, mechanical properties, elastic recovery, biobased polymers

## Abstract

The aim of this paper is to extend knowledge on biobased poly(butylene furanoate)–*block*–poly (ethylene oxide) (PBF-b-PEO) copolymers’ performance by studying the effect of the PEO segment’s molecular weight on the microstructure and materials behavior. As crystallization ability of PEO depends on its molecular weight, the idea was to use two PEO segment lengths, expecting that the longer one would be able to crystallize affecting the phase separation in copolymers, thus affecting their mechanical performance, including elasticity. Two series of PBF-block-PEOs with the PEO segments of 1000 and 2000 g/mol and different PBF/PEO segment ratios were synthesized by polycondensation in melt, injection molded to confirm their processability, and subjected to characterization by NMR, FTIR, DSC, DMTA, WAXS, TGA, and mechanical parameters. Indeed, the PEO2000 segment not only supported the crystallization of the PBF segments in copolymers, but at contents at least 50 wt % is getting crystallizable in the low temperature range, which results in the microstructure development and affects the mechanical properties. While the improvement in the phase separation slightly reduces the copolymers’ ability to deformation, it is beneficial for the elastic recovery of the materials. The investigations were performed on the injection molded samples reflecting the macroscopic properties of the bulk materials.

## 1. Introduction

Research on the synthesis and characterization of polymer materials based on biomass-derived monomers, possibly biodegradable and not harmful for the environment, is currently one of the most important trends in the materials science development. It is mainly focused on looking for green alternatives for the most commercially relevant and consumable polymers like polyolefins, polyamides, polyurethanes, or polyesters, among others [1,2,3,4,5]. The constantly growing eco-awareness of the customers and their expectations for more sustainable materials encourage the plastic industry and academia to join together on the research projects for more effective development and commercialization of the bioplastics [6].

Furan polyesters and copolyesters are the best example of the biobased polymer group that has been extensively investigated over the last few years. Although the main monomer, plant-derived 2,5-furandicarboxylic acid (FDCA), has been known since the 1940s, when first patents for its application were claimed [7], it has gained an importance recently for the synthesis of the green counterparts of the petroleum-based aromatic polyesters. Indeed, poly(ethylene furanoate) (PEF), poly(butylene furanoate) (PBF), and poly(trimethylene furanoate) (PTF), may be obtained as fully biobased, and due to their semicrystalline nature, processability, thermal parameters, and mechanical strength are classified as high-performance polymer materials [8,9,10] with a wide variety of possible applications in food and beverages packaging, textile, carpets, electronics, or automotive, among others. Although PEF and PTF homopolymers are very close to commercialization [11,12], PBF, due to good combination of thermal and mechanical properties with a relatively fast crystallization (if compared to PEF and PTF), is the most suitable as the building block to copolymers in order to extend applicability of the FDCA-based materials. Such features are particularly important in development of thermoplastic copolyester elastomers (TPEE), which, in fact, are block copolymers, consisting of alternately arranged rigid and flexible segments, and their specific performance results from a phase micro- and nanoseparation [13]. This is driven by crystallization ability of the rigid segments forming crystalline nanodomains of a hard phase, while the flexible segments should stay amorphous forming a soft phase and providing elasticity to the material. Considering crystallizability of PBF, Papageorgiou et al. reported that during cooling from melt PBF crystallizes within the temperature range of 150 to 60 °C, depending on cooling rate, with high nucleation density, and the same temperature range was observed for the cold crystallization effect during subsequent heating [14]. The effect of phase separation in the microstructure and a significant modification of the mechanical behavior towards elastomers was observed in several studies, in which PBF was used as the rigid segment, while the flexible segment was poly (tetramethylene glycol) (PTMG) [15] (analogous to commercially available PBT/PTMG), diglycolate [16], or dimerized fatty acids [17].

Recently a few studies on PBF block copolymers have been published, in which poly (ethylene oxide) (PEO) or poly (propylene oxide) (PPO) were used as the co-monomers [18,19,20]. The special interest in these building blocks lies in their non-toxicity and biocompatibility [21], that extends the potential applications of the biobased copolymers also for biomedical purposes. Low glass transition temperature, high mobility of chains, and amorphous or semicrystalline nature, depending on the molecular weight, make these polyethers ideal candidates as the flexible segments. What is also important is that PEO is commercially available as the biobased product by Acme–Hardesty Co., received from the fibrous wastes that remain after sugar cane stalks processing [22]. PEO was previously used for the synthesis of block copolymers combining with petroleum-based aromatic polyesters like poly (ethylene terephthalate) [23,24], poly (butylene terephthalate) [25,26,27], and poly (trimetylene terephthalate) [28,29,30,31,32]. In general the materials revealed the tendency to phase separation depending on the segments’ ratio, modified but acceptable thermal and mechanical properties as compared to the polyester homopolymer, and particularly interesting CO_2_ permeability and selectivity for gas separating membranes’ applications [25,33]. The PEO/PBT multi-block copolymers with different molecular weights of PEO as well as PEO to PBT segments’ ratio have been also commercialized under the trade name PolyActive^TM^, and are used for manufacturing of the 3D–printed bio-scaffolds [34] as well as biodegradable drug delivery systems and implants [12].

As mentioned above, the series of PBF/PEO copolymers containing the PEO segment with the molecular weight of *M*_n_ = 1000 g/mol and different segments’ ratio have been already characterized confirming their segmented structure and semicrystalline nature. However, the results concerning thermal parameters are incoherent. Sousa et al. performed extensive thermal studies of copolymers containing 14 to 68 mol% of the PEO segment using fast scanning calorimetry technique among others. A severe decrease in melting and glass transition temperatures was observed when compared to PBF homopolymer, even for the smallest content of the soft segment (107 vs. 171 °C and −35 vs. 71 °C for *T*_m_ and *T*_g_ respectively) [18]. *T*_m_ values for copolymers with higher PEO content were even lower and varied 30–41 °C, which was explained as melting PEO crystalline domains instead of PBF. Concurrently, the glass transition effects of PBF amorphous phase were not detectable on DSC thermograms. Significantly different thermal parameters were reported by Hu et al. for PBF/PEO copolymers with 10 to 60 wt % of PEO [19]. For this series of materials, the *T*_m_ values decreased gradually from 167 to ca. 122 °C and *T*_g_ values from 35 to −34 °C along with the PEO content increase, which makes the copolymers much more applicable, though the crystallinity of copolymers was relatively low. The materials revealed varied mechanical behavior towards elastomeric features, and susceptibility to degradation, particularly in alkaline conditions. Therefore, the aim of this paper is to extend the knowledge on biobased PBF/PEO copolymers performance by studying the influence of PEO segment length on the resulting microstructure and materials behavior. As a crystallization ability of poly (ethylene oxide) depends on its molecular weight, the idea was to use two different PEO segment lengths, expecting that the longer one would be able to crystallize developing or supporting the phase separation in copolymers, and to affect the mechanical performance of the materials as well. Thus, two series of poly(butylene furanoate)–*block–*poly(ethylene oxide) (PBF-*b*-PEO) with the PEO segments of 1000 and 2000 g/mol in molecular weight were synthesized by polycondensation in melt, injection molded to confirm their processability, and subjected to characterization. The chemical and crystalline structure, thermal properties and mechanical performance, especially elastic properties, of PBF-*b*-PEO block copolymers are compared and discussed.

## 2. Materials and Methods

### 2.1. Materials

2,5–Furandicarboxylic acid dimethyl ester (DMFDCA), 99% in purity, was supplied by Matrix Fine Chemicals, Switzerland. Renewable 1,4-butanediol (bio-BD) was kindly supplied by BASF SE, Germany. Poly (ethylene oxide)s, BioUltra 1000, and 2000 g/mol (PEO1000 and PEO2000) were purchased from Sigma-Aldrich Chemie GmbH, Steinheim, Germany. The other reagents—tetrabuthyl orthotitanate, Ti(OBu)_4_, (Fluka) the catalyst, Irganox 1010 (BASF, Ludwigshafen, Germany), the antioxidant, deuterated chloroform (CDCl_3_), trifluoroacetic acid (CF_3_COOD), and phenol/1,1,2,2-tetrachloroethane mixture (60/40 wt %)—were purchased from Sigma Aldrich and used as received.

### 2.2. Synthesis of PBF and PBF-Block-PEO Copolymers

The investigated materials were obtained via two-stage melt polycondensation method. A detailed procedure of furan-based polyesters’ synthesis using a 1 dm^3^ in volume chemical reactor has been described previously in [17,35,36]. The PBF segment was synthesized by a transesterification reaction between DMFDCA and bio-BD, catalyzed by Ti(OBu)_4_ (0.25% in relation to the ester in total)_,_ and methanol was released as a by-product. The ester to diol molar ratio was 1:2. The reaction proceeded in the temperature range 150 to 160 °C within about 2 h. When ~80% of the theoretical amount of methanol was distilled out, the reaction temperature was gradually raised, and an appropriate amount of PEO (depending on the PBF to PEO segment ratio), together with the second part of the catalyst and antioxidant (0.5 wt % in relation to the copolymer final mass), were added into the reaction mixture. The second stage, melt polycondensation, was carried out under reduced pressure (25–30 Pa), in the temperature range of 230 to 240 °C, and monitored via gradual increase of the stirrer torque. The same process parameters were applied for all synthesized materials, and the whole reaction lasted 5–6 h in total. Finally, the homopolymer/copolymer melt was extruded from the reactor under the N_2_ pressure, and then granulated. The material output was ~150–160 g, enough to subject the copolymers to processing by injection molding.

This method two series of PBF-*b*-PEO copolymers containing 80, 65, 60, and 35 wt % of the PBF rigid segment and PEO1000 or PEO2000 flexible segment have been prepared and subjected to the characterization. In the text the materials are denoted as PBF-PEO_1000_ or PBF-PEO_2000_ with a relevant number referring to the rigid segment wt % content.

The dog-bone-shaped samples (according to ISO 37:2005, type 3) for further characterization, including the tensile tests, were prepared using the injection molding machine Boy 150 (Dr Boy, Neustadt—Fernthal, Germany). Granulates were carefully dried before processing at 80 °C for 6 h. The molding temperature varied between 170 and 220 °C (increasing along with PBF content), the injection pressure was 50 MPa, holding down pressure was 25 MPa, and the temperature of mold was 30 °C.

### 2.3. Characterization Methods

The intrinsic viscosities [η] of all investigated samples were determined using an Ubbelohde viscometer (type Ic, *K* = 0.03294) in a single-point method. The 0.5 g/dL polymer solutions in phenol/1,1,2,2-tetrachloroethane (60/40 wt %) were tested at 30 °C.

For determination of the number-average molecular weight (*M*_n_) and molecular weight distribution (Ð) of materials, the size-exclusion chromatography (SEC) measurements were performed at 40 °C using Waters GPC instrument (Waters Corporation, Milford, MA, USA) with two PSS (PFG-lin-XL, 7 μm, 8 × 300 mm) columns. 1,1,1,3,3,3-hexafluoroisopropanol (HFIP) with potassium trifluoroacetate was used as the eluent, and PMMA standards for calibration. The measurements were done for the PBF homopolymer and copolymers containing 50 wt % of PEO1000 or PEO2000.

The ATR-FTIR spectra were obtained using Tensor-27 (Brucker, Ettlingen, Germany) spectrophotometer equipped with a germanium crystal ATR accessory. The spectra were recorded in a wave number range of 4000 to 600 cm^−1^ and normalized.

The ^1^H-NMR analysis of the copolymer’s chemical structure was performed using Bruker 400 MHz spectrometer. All samples in a granulate form were first extracted in methanol on a Soxhlet extractor for 72 h each. CF_3_COOD/lockD_2_O or CDCl3 were used as solvents (depending on the PBF content), and tetramethylsilane (TMS) as an internal reference. The integral intensities of the characteristic peaks were used to calculate the real fractions of the PEO segments in synthesized copolymers applying Equation (1):

(1) $$W_{PEO}\left(wt\;\%\right)=\;\frac{44\;\left(\frac{I_{d}}{4}\right)}{44\;\left(\frac{I_{d}}{4}\right)+\;210\;\left(\frac{I_{b}}{4}\right)}100\%$$

where *I_d_* is the integral intensity of the signal at 3.64 ppm, which arised from the aliphatic protons of methylene groups in the PEO backbone, and *I_b_* refers to the aliphatic methylene protons in buthylene unit of the PBF segment. The values indicate the molecular weights of the following sequence: 44 refers to the PEO sequence in the PEO segment, and 210 refers to the PBF repeating units. The mole fractions of the flexible segment were calculated using Equation (2):

(2) $$W_{PEO}\left(mol\;\%\right)=\;\frac{226\;\left(\frac{I_{d}}{85.27}\right)}{226\;\left(\frac{I_{d}}{85.27}\right)+\;210\;\left(\frac{I_{b}}{10}\right)}100\%$$

where 226 is the molecular mass of the whole PEO flexible segment, whereas 85.27 and 10 are the numbers of protons in the corresponding repeating units.

For thermal analysis the differential scanning calorimetry (DSC, Q100 TA Instruments, New Castle, DE, USA) technique was used. The materials were studied in heating–cooling–heating cycles with the standard rate of 10 °C/min, and the temperature range of −60 to 250 °C. The cooling and second heating scans were used to determine the melting and crystallization/cold crystallization temperatures, as the maximum of endothermic or exothermic peaks in the thermographs. The glass transition temperatures (*T*_g_), in turn, were taken as the midpoint of the heat capacity (Δ*c*_p_) increment. The enthalpies of melting (Δ*H*_m_) and crystallization (Δ*H*_c_) were determined by the integration of the normalized area of the peaks associated with a specified transition. The crystallinity degree (*x*_c_) of the investigated materials was calculated as the ratio of the melting enthalpy with the enthalpy of the cold crystallization subtracted (if observed) to the heat of fusion of fully crystalline polyester (Δ*H*^0^_m_). For PBF Δ*H*^0^_m_=129 J/g [14].

The thermal stability of the PBF-*block*-PEO copolymers was studied using thermogravimetric analysis (TGA 92-16.18 SETARAM Instrumentation). The measurements were carried out with the heating rate of 10 °C/min up to 600 °C in both air and nitrogen atmospheres.

Dynamic-mechanical thermal properties (DMTA) were performed on DMA Q800 thermal analyzer (TA Instruments), operated in multifrequency strain mode. The measurements were performed in the temperature range of −100 to 120 °C at a constant frequency of 1 Hz and heating rate of 3 °C/min.

The crystalline structure of the injection molded samples was investigated by wide angle X-ray diffraction (WAXS, X’PERT PANALYTICAL) using CuK_α_ radiation lamp at 0.154 nm wavelength. The samples were scanned in the scattering angle range of 0 to 40° with 0.1° step. The obtained spectra were edited and analyzed using WAXSFIT computer program [37], and the crystallinity degrees (X_c_) of copolymers were calculated as the ratio of crystalline peaks integrated area to the total X-ray spectrum area.

The mechanical performance of copolymers was studied in tensile tests using the universal testing machine (Autograph AG-Xplus, Shimadzu, Kyoto, Japan), equipped with a video extensometer TRViewX and 1kN load cell, according to ISO 527-1,2: 2012. The samples with a total length of 60 mm, a rectangular cross section of 2 × 4 mm^2^, and a gage length of 20 mm, were first deformed up to 1% of strain at the speed of 1 mm/min in order to calculate the tensile modulus E, then the speed was raised to 100 mm/min. All tests were performed at room temperature. A minimum of six tests were done for each material. The average values of tensile strength, stress at break, stress at yield (if observed), elongation at break and tensile modulus were calculated from the stress–strain curves.

The same equipment was used in cyclic loading–unloading tensile tests in order to evaluate the elastic properties of PBF-b-PEO copolymers. This procedure was described in details in [36], and in general a sample was deformed until reaching a pre-defined value of strain, then the tensile force was released to the zero value, and the same cycles were repeated reaching higher and higher levels of strain, i.e., 5, 10, 25, 50, 100, 200, and 400%. A deformation speed was 100 mm/min. After every cycle of loading and unloading the length of the sample measuring section was measured and a permanent set or, in other words, an ability to elastic recovery was determined.

The Shore hardness of investigated materials was measured on durometer type D (Zwick) after 15s of loading. The density of samples was determined at 23 °C on the hydrostatic balance (Radwag WPE 600C, Radom, Poland), calibrated according to the standards with known density.

## 3. Results and Discussion

### 3.1. Macromolecular Structure of PBF-Block-PEO Copolymers

**Figure 1:**
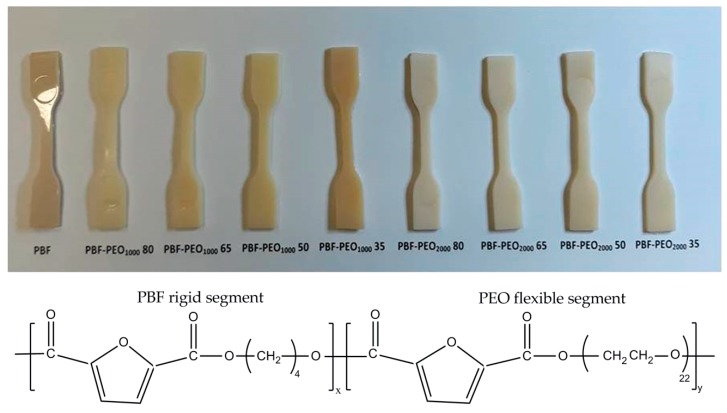
Chemical structure of PBF-*block*-PEO copolymers (x,y–degree of polymerization) and the photo of injection molded samples.

Two series of PBF–block–PEO copolymers, in which PBF is defined as the rigid and PEO as the flexible segment, varied in the PEO segment’ molecular weight, and PBF to PEO ratio, as well as the PBF homopolymer, were successfully synthesized by a conventional two-step polycondensation in melt, starting from FDCA dimethyl ester and reasonable excess of bio-BD (1:2), under relatively mild process conditions. The changes in the copolymers’ composition were controlled via changes in a degree of polymerization, DP_x_, of the PBF segment (Table 1). The intrinsic viscosity numbers [η], determined at 30 °C, were: 0.87 for the homopolymer, and 0.97 to 1.27 dL/g for copolymers with increasing PEO content, regardless its molecular weight. These values correspond to the results reported in [19]. Moreover, the materials were characterized by the following molecular weights (*M*_n_), i.e., 34,100 for PBF homopolymer (Ð = 1.8) as well as 56,400 (Ð = 1.9) and 69,000 (Ð = 2.0) for PBF-PEO_1000_50 and PBF-PEO_2000_50 samples, respectively. The materials are simple in processing by injection molding (Figure 1). No shrinkage effect was observed after processing, which makes them suitable for the thermally molded precise elements.

**Figure 2:**
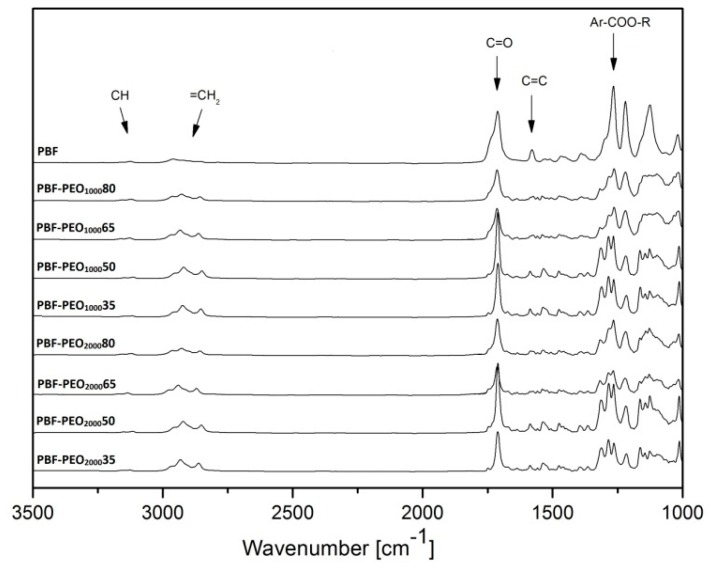
ATR–FTIR analysis of PBF-*block*-PEO copolymers with PEO1000 and PEO2000 segments.

The chemical structure and the real composition of PBF-*b*-PEO copolymers were evaluated based on FTIR and ^1^H-NMR results. The ATR-FTIR spectra, presented in Figure 2, show the absorption peaks characteristic for furan heterocycles and for PEO bonds with varied intensities. The PBF homopolymer reveals typical reflections at 1578–1580 cm^−1^, attributed to the C=C stretching bonds, and at 3127 and 3157 cm^−1^, arised from the C–H stretching bonds in the furan rings. Furthermore the breathing peaks observed at around 1019–1021 cm^−1^, and the bending motions peaks at around 966, 860–861, and 763–764 cm^−1^ are associated with 2,5-disubstituted furan heterocycles. Similar signals, but less intense, are observed at the copolymers’ spectra due to decreasing concentration of the furan moieties. In turn, the reflections given by the ester groups, detected near 1721 cm^−1^ and 1260–1268 cm^−1^ (stretching vibration of C=O and C–O, respectively), are relatively strong for all investigated materials. The signals attributed to PEO segments are recognized as the reflections at 2868–2870 cm^−1^, arising from the antisymmetric and symmetric stretching of C–H in CH_2_ groups of PEO chain, and near 1280 and 1362 cm^−1^, attributed to C–O–C stretching vibrations. The absorption peak at 2949 cm^−1^ also refers to C–H bond in CH_2_ groups in bio-BD moiety. There is a lack of reflections at the 3378–3400 cm^−1^ wavenumber range, attributed to the stretching vibrations of –OH bonds, present in the unreacted PEO. This fact confirms a successful effect of the polycondensation process, where the PEO segments have been fully reacted with PBF oligomeric segments.

**Figure 3:**
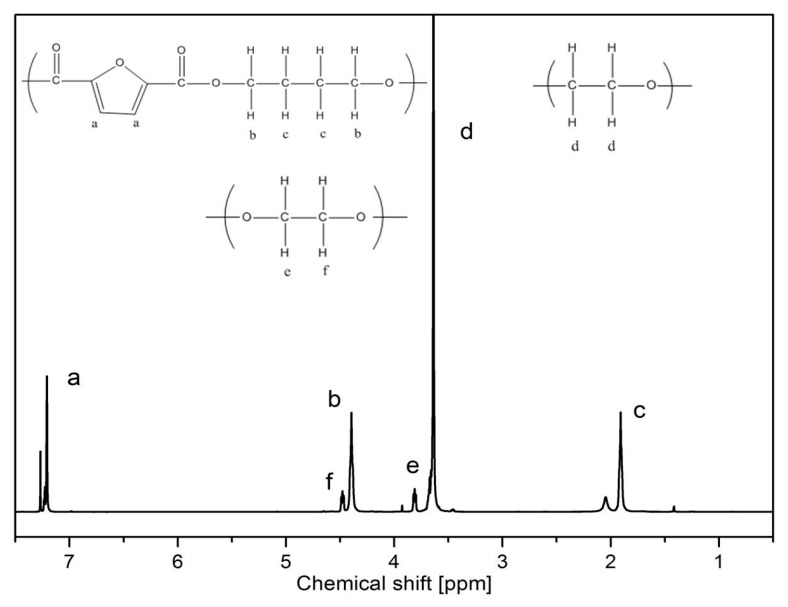
The representative ^1^H NMR spectrum of PBF-PEO_1000_50 copolymer sample.

More details about the real chemical structure and composition of the copolymers are provided by ^1^H-NMR analysis. The representative spectrum of the PBF-PEO_1000_50 copolymer sample is presented in Figure 3, and the results for other copolymers are collected in Table 1. The peak positions were taken from the PBF-PEO_1000_ copolymers’ spectra, and positions for PBF-PEO_2000_ copolymers are presented in brackets.

In general, the obtained ^1^H-NMR results confirm the expected multiblocked structure of the synthesized copolymers with randomly distributed rigid and flexible segments. Starting from left the resonance at 7.21 ppm (peak a (7.21)) corresponds to 2H aromatic protons of the furan ring, and the resonances detected at 4.40 ppm (peak b (4.40); triplet, 4H, –OCH_2_–) and 1.91 ppm (peak c (1.91); multiplet, 4H, –(CH_2_)_2_–) refer to the aliphatic methylene protons in buthylene unit of the PBF segment. Additionally, the peak b indicates the CH_2_ group adjacent to the carbonyl linkage of the ester group, which confirms a transesterification reaction between the hydroxyl end groups of PEO and the ester groups in PBF segment during the synthesis. In turn, the resonances at 4.48 ppm (peak f (4.47); triplet, 4H, –OCH_2_–), and 3.81 ppm (peak e (3.82); triplet, 4H, –OCH_2_–) may be assigned to the methylene protons of the PEO chain, wherein one in connected via the ester bond to the furanoate unit (f), and second connects the PEO backbone (e). The most intense resonance at 3.64 ppm (peak d (3.64); 84H, 21 × CH_2_)_2_) corresponds to the backbone of the aliphatic protons of methylene groups in the PEO segment. The intensity of this peak varies depending on the PEO segments’ length in the copolymers. As seen in Table 1 the integral intensities of signals d (*I*_d_) and e (*I*_e_), corresponding to the presence of the flexible segment, are changing the most due to their different content in copolymers. The obtained results strictly correspond to the ^1^H-NMR results reported for the similar PBF/PEO or PBF/PPO copolymers [18,19,20]. The calculated real contents of the rigid and flexible units are also very close to those theoretically calculated, what confirms once again the expected segmented structure of the copolymers.

### 3.2. Effect of the PEO Segment Length on Microstructure and Thermal Properties of PBF-Block-PEO Copolymers

**Figure 4:**
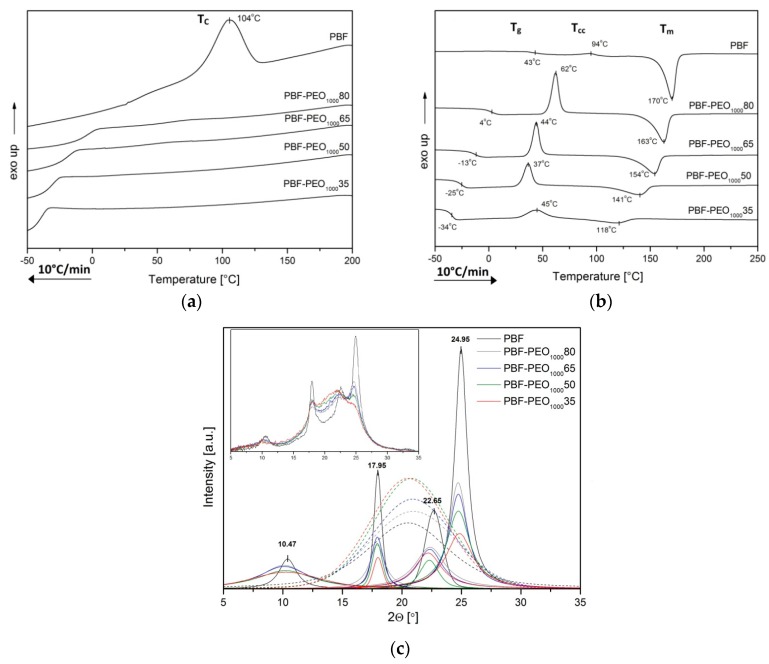
The DSC scans of cooling (**a**) and heating (**b**) as well as the WAXS analysis (**c**) of PBF–*block-*PEO_1000_ copolymers.

**Figure 5:**
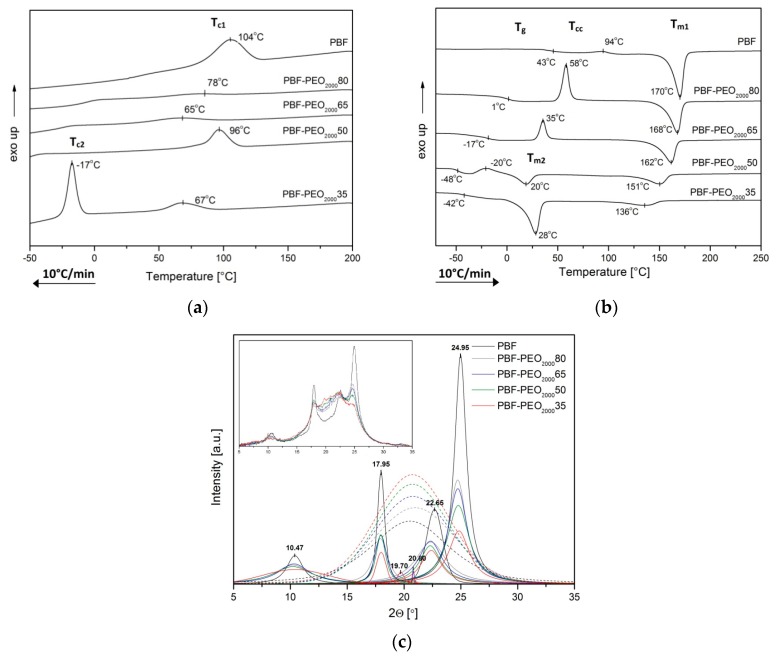
The DSC scans of cooling (**a**) and heating (**b**) as well as the WAXS analysis after WAXFIT editing (**c**) (the raw data were added as inset picture) of PBF–PEO_2000_ copolymers.

The literature survey on the poly(ether–ester) block copolymers with poly(ethylene oxide) provided the evidence that such materials, due to a thermodynamic immiscibility of the constituents, reveal the tendency to the phase separation, in which polyester segments form a semicrystalline hard phase, whereas PEO stays amorphous forming a PEO-rich soft phase. Such microstructure results in an elastomer-like behavior, and due to its thermoreversible character, the copolymers may be processed as typical thermoplastics. The new group of furan polyesters is, however, less crystallizable than the conventional petroleum based polyesters, which also affects the effectiveness of the phase separation in copolymers. As the crystallization ability of PEO depends on its molecular weight (it is becoming crystallizable for the *M*_w_ above 600 g/mol [38]), it is expected that the PEO segment length should influence the crystallization ability, phase transition temperatures, and resulted microstructure of the copolymers. To study all these effects, the copolymers with PEO segments of 1000 and 2000 g/mol, and different PBF to PEO ratio have been investigated by DSC, WAXS, and DMTA techniques, and compared. The characteristic thermal and structural parameters are summarized in Table 2, while the DSC thermograms and XRD patterns are presented in Figure 4 and Figure 5.

Although the PBF homopolymer reveals a clear crystallization peak, *T*_c_, at 104 °C as observed on the standard cooling scans of investigated materials, the PBF-PEO_1000_ copolymers do not show any effects of crystallization in neither PBF nor PEO segments. In turn, relatively strong glass transition effects are observed in sub-ambient temperature range (Figure 4a). However, when the subsequent heating scans are analyzed, the cold crystallization peaks are easily detected for all samples, as well as the melting and glass transition effects, which confirms the semicrystalline nature of the copolymers (Figure 4b). These observations are contrary to the crystallization results reported by Hu [19], where an incorporation of PEO1000 segments supported the melt crystallization process in all investigated copolymers, but correspond to the FSC studies by Sousa [18]. It was concluded that the PBF/PEO copolymers’ crystallization rate is slower than that of the neat PBF, and no crystallization or melting peaks were observed for varied cooling rates. Indeed, from our DSC results one can say that, in the PBF-PEO_1000_ copolymers both segments are miscible at the molecular scale, which results in existing only one (mixed) phase in the copolymers’ microstructure. It is confirmed by only one glass transition effect observed in the cooling scans of materials with different PBF to PEO segments’ ratio as well as the lack of melt crystallization effects (Figure 4a). The *T*_g_ values of the samples are shifted to the lower temperature range (from 4 to −34 °C), when compared to the neat PBF, along with the PEO1000 content. It provides the evidence for improved macromolecular flexibility by the incorporation of the PEO sequences within the PBF oligomers, but also that this physical transition is governed by the amount of the flexible segment. It is also confirmed by increasing values of the heat capacity at *T*_g_ (Δ*C*_p_) in the copolymers (Table 2). In the condensed state, during the subsequent heating cycle (Figure 4b), the copolymers’ microstructure remains homogeneous until the glass transition range is exceeded, and the cold crystallization of the PBF segments occurs. This transition becomes a driving force of the microstructure separation into two phases: the PBF crystalline hard phase and the soft one, being a mixture of two segments, but dominated by the PEO1000, which stays amorphous. Of course, the *T*_cc_ values of copolymers are decreasing with the flexible segment content due to its plasticizing effect on the rigid segment folding, whereas the cold crystallization enthalpies (Δ*H*_cc_) depend on the amount of crystallizable PBF blocks. Under further heating the crystalline nanodomains of the hard phase are getting melt, and the observable decrease of *T*_m2_ values of copolymers as well as their enthalpies (Δ*H*_m2_) are also associated with decrease of the PBF content. However, additionally, it may result from not perfectly developed crystalline structure, disturbed by the presence of chemically bonded PEO segments or larger interfacial effects on the melting transitions. Actually, the presence of only one crystalline form in the copolymers’ microstructure is confirmed by the WAXS diffractograms displayed in Figure 4c. When separated to the diffraction peaks and amorphous background, and then compared to the pattern of the semicrystalline PBF sample, it is clear that copolymers reveal only the characteristic crystalline peaks with the scattering angle positions very close to those of the PBF (i.e., 10.4; 18; 22.7, and 25° of 2θ). At the same time the reflections are losing their slender shape along with the PEO content, while the amorphous phase background gains in the intensity, which is a consequence of decreasing crystalline phase’s content in the copolymers. Indeed, the crystallinity degrees (*X*_c_), calculated from WAXS, vary from 55% for the neat PBF to 38% for the copolymer containing only 35 wt % of the furan–ester segment. Note that the similar thermal behavior and parameters were reported for PBF copolymers with dimerized fatty acid as the flexible segment [17]. However, in the current study, the *T*_g_ and *T*_cc_ temperatures are slightly lower, which provides the evidence for improved chain mobility due to the higher length of the PEO segment (1000 vs. 570 g/mol of the fatty acid diol).

Considering the DSC thermograms of PBF-PEO_2000_ copolymers, the differences in their thermal behavior due to increased PEO segment length become clear for materials containing 50 and 65 wt % of the flexible segment. When the PBF segment is predominant, the copolymers reveal analogous phase transitions with *T*_g_ and *T*_cc_ values slightly shifted to lower temperatures, whereas *T*_m_ values are higher if compared to PBF-PEO_1000_ samples (Figure 5, Table 2). Moreover, the very weak melt crystallization effects, in temperature range related to T_c_ of the neat PBF, may be detected in the cooling traces (Figure 5a). These observations suggest that the macromolecules gain in flexibility with a longer length of the PEO segment, which initiates and drives the phase separation by forming the crystalline nanodomains. In the copolymers’ microstructure, a PBF-rich continuous phase dominates. An increase of the melting temperatures also indicates higher quality of the crystalline phase. However, when the PEO2000 content is increasing, its molecular weight enables the crystallization of the soft segments, and the evolution of the copolymers’ microstructure is evident when comparing the DSC scans of PBF-PEO_2000_50 and PBF-PEO_2000_65 samples. In case of the first one, when cooling from the melt, only one clear exothermal effect is observed, and its temperature suggests the PBF segment crystallization. However, during a subsequent heating the low-temperature cold crystallization peak is observed, and two melting temperatures *T*_m1_ and *T*_m2_ (Figure 5b). For the copolymer with the highest PEO2000 content, in turn, two melt crystallization effects (*T*_c1_ and *T*_c2_) can be distinguished, and two melting peaks on the 2nd heating traces. There are no doubts, that all thermal effects observed in the low temperature region are strictly related to the PEO segment induced crystallization. These crystallization/melting peaks are gaining in the intensity along with the segment content. At the same time an increased molecular weight of the flexible segment entails an increase of the polymerization degree (DP_x_) of the PBF segment. It results in extension of the length of the rigid segments in the polymer chains as well, which together with the improved molecular mobility and diffusion, enables their folding. That would explain the appearance of the PBF melt crystallization effects in PBF-PEO_2000_ samples. Consequently, two kinds of crystalline phases with different *T*_m_ coexist in the copolymers’ microstructure. In turn, only one glass transition region is detected with *T*_g_ values significantly shifted to the low temperature region (−42 to −48 °C) when compared to the both PBF-PEO_1000_ or other PBF-PEO_2000_ copolymers. On one hand, it indicates that due to the chemical bonds between the rigid and flexible segments they are not able to separate to two neat phases given two *T*_g_ effects. On the other hand, such a big drop in *T*_g_ values suggests that for the materials containing 50 wt % and above of the PEO2000 a phase inversion occurs, and the PEO-rich soft amorphous phase is getting a continuous phase with dispersed crystalline nanodomains of the PBF hard phase. A decrease of the Δ*C*_p_ values at *T*_g_ also suggests an improved phase separation in the copolymers due to the PEO assigned crystals forming.

The reorganization in crystalline structure of the materials should be reflected by the WAXS diffractograms. According to the literature, the PEO crystallites give two characteristic peaks at 19.2 and 23.2° scattering angles [39]. When the PBF-PEO_2000_ copolymers’ patterns are analyzed, four main peaks corresponding to PBF crystalline reflections can be identified (Figure 5c), and two, relatively weak, and noticeable only for the copolymers, peaks at 19.7 and 20.75° (actually the 19.7° position peak is detectable only for PBF-PEO_2000_ 35 sample). Note that the investigated materials are quite complex multiphase systems; moreover, the WAXS measurements were performed at room temperature, whereas the melting of the PEO assigned crystalline phase was detected within 20–28 °C (Figure 5b). This might suggest that first the low-temperature formed crystalline phase is affected/defected due to the presence of the PBF assigned crystals, which would explain why the peak positions do not exactly match the PEO related peak positions. Second, in the room temperature the same crystalline phase could partly melt, also affecting the patterns. Nevertheless, the crystallinity degree, calculated from the WAXS, is higher for the PBF-PEO_2000_ copolymers with the largest PEO segment contents than the comparable PBF-PEO_1000_ materials (Table 2). Thus, it can be concluded that both DSC and WAXS results provide clear evidence that not only the PBF to PEO segment ratio, but also the molecular weight of the PEO flexible segment significantly affect the microstructure and phase separation in PBF-*block*-PEO copolymers.

**Figure 6:**
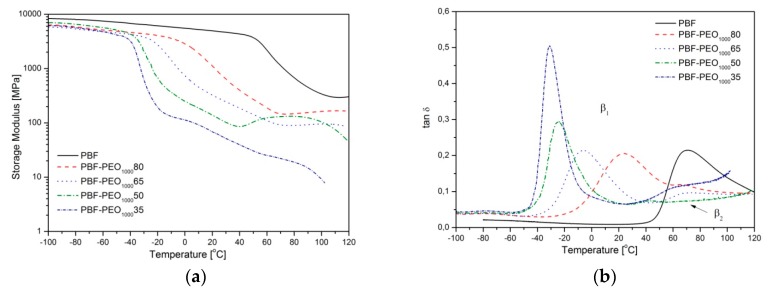

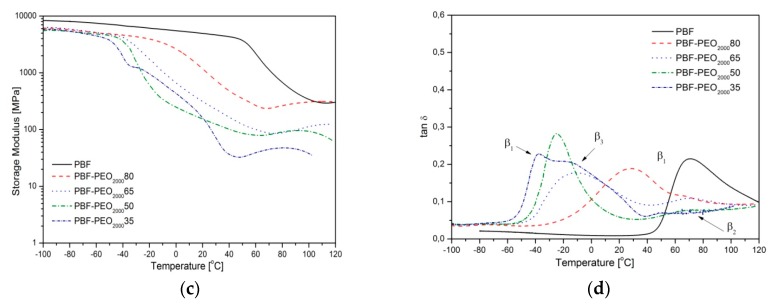
The dynamic-mechanical thermal properties (DMTA) analysis of PBF–block–PEO copolymers with PEO1000 and PEO2000: the storage modulus *E*’ (**a**,**c**) and tanδ (**b**,**d**) as a function of temperature.

A thermo-mechanical behavior of the investigated materials, studied by the DMTA technique, only supports these conclusions. Changes of the storage moduli (*E*’) as well as the loss factor (tanδ) within the −100 to 120 °C temperature range are presented in Figure 6a–d. The PBF homopolymer, as the reference, reveals a clear relaxation, β_1_, on tanδ curve corresponding to a drop at the storage modulus profile, and attributed to the glass transition of its amorphous phase. In the case of the PBF-PEO_1000_ copolymers’ samples, the main relaxation peaks β_1_ are successively shifted towards the lower temperatures with increasing flexible segment content, which is also reflected by the observed decreasing *E*’ modulus values. These findings confirm once again an improved macromolecular mobility due to an incorporation of amorphous and highly flexible PEO segments. Under the heating; however, the second relaxation effect, β_2_, is detected on tanδ profile within the temperature range of 40 to 70 °C, and accompanied by a visible shoulder on *E*’ curves. As confirmed by the WAXS analysis, it is attributed to the thermally and mechanically induced cold crystallization of the PBF segment in the copolymers’ microstructure, also observed on the DSC heating termograms. Considering the practical aspects of the copolymers’ performance, note that if the loss factor is an indicator of the material’s ability to energy dissipation, the increasing content of the PEO1000 segment improves the copolymers’ damping capability. Particularly the PBF-PEO_1000_50 and 35 samples reveal the highest β_1_ relaxation values and intensities, which is another advantage of their specific heterogeneous microstructure. Additionally, the same copolymers at the room temperature, are in the elastic state, which should also affect their mechanical performance.

In general, similar effects are observed for the copolymers with the PEO2000, until its content makes the flexible segment crystallizable. This is clearly visible by the variations in the *E*’ and tanδ profiles of the PBF-PEO_2000_35 sample (Figure 6c,d). What can be seen on the thermograms is: the relaxation related to *T*_g_ at approximately −45 °C (β_1_), then the shoulder within approximately −20 to 0 °C temperature range (β_3_), which may suggest a cold crystallization of PEO segments, another drop at approximately 0 to 30 °C, corresponding to melting of PEO assigned crystalline structure, and further relaxation within 40° to 70 °C, also observed for other copolymers, and attributed to PBF segment crystallization (β_2_). Not all of these relaxations were detectable on the DSC scan; however, DMTA analysis is considered as more sensitive for the phase transitions in the polymers. An increase of the crystalline nanodomains in the microstructure also disturbs its ability to the vibration energy dissipation as indicated by a drop of tan δ maximum. This is likely due to the reduction of a free volume and flexibility of the continuous soft phase. Thus, although a development in the copolymers’ microstructure induced by the crystallization of the PEO soft segments seems to be interesting from the scientific point of view, it makes this microstructure very unstable in fact, particularly within approximately −20 to 30 °C temperature range. In practice, it means that the materials’ performance will be affected by the working temperature even more than the typical thermoplastics, which limits their application profile. Alternatively additional treatment like annealing would be needed to allow the crystalline structure to get fully formed and stabilized.

**Figure 7:**
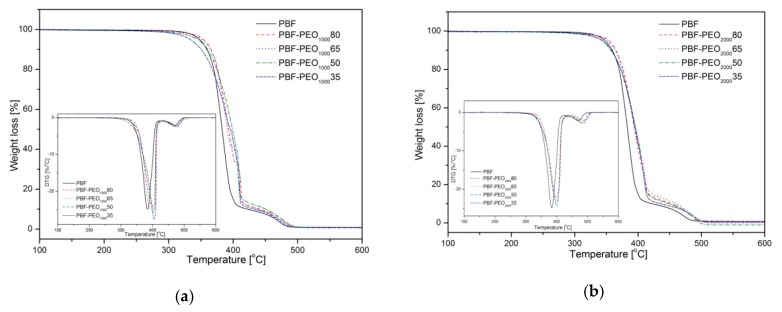
TGA thermograms of PBF homopolymer and PBF-block-PEO1000 (**a**) and PBF-block-PEO2000 (**b**) copolymers under thermo-oxidative atmosphere.

The thermal stability, particularly in the thermo-oxidative atmosphere, is also an important factor in the characterization of PBF-*block*-PEO copolymers due to their suitability for the thermal processing, but has not been analyzed by other authors. The TGA thermograms for both series of materials are presented in Figure 7a,b, and the degradation temperatures were collected in Table 2. In general, the decomposition of the furan polyesters proceeds in two steps. First, the ester linkages are subjected to the scission forming carbonyl-end groups and vinyl esters, then the oxygen atoms react with the methylene groups in diester linkage forming hydroperoxides [40]. Such decomposition profiles were observed for the furan–polyester copolymers with the dimerized fatty acid flexible segment, regardless of which glycol was used to synthesize the rigid segment [17,35,36,41]. Also, in most cases, the presence of the fatty acids improved the stability of the copolymers. In currently investigated materials it is clear, that the PBF-*block*-PEO copolymers reveal the thermo-oxidative stability at the same level as the PBF homopolymer, also an increase of 10–15 °C in *T*_decMAX_ is observed. It means that under processing conditions undesired degradation reactions should not occur in materials structure. More detailed analysis of the TGA results shows that the decomposition temperatures vary with the PBF to PEO segment ratio. At the 5% of the weight loss (*T*_dec 5%_) the copolymers with the highest content of PBF segments reveal an improved stability compared to neat PBF, but along with the PEO segment content the *T*_dec 5%_ values are slightly decreasing. Furthermore, more fluctuations, particularly in the first step of degradation profile, are observed for PBF- PEO_1000_ samples. This may be related to a higher concentration of the ester/ether groups in the copolymers’ macromolecules with the PEO segments with lower molecular weight, which in turn are subjected to random scission intensified by the oxygen atoms. According to the literature PEO is also susceptible to a free radical oxidative attack, and during the degradation some volatile compounds can be released [42]. Slightly better performance of the PBF-PEO_2000_ copolymers may be explained, in turn, by enhanced interactions between PBF and PEO segments, as suggested in [18].

### 3.3. The Effect of the PEO Segment Length on Mechanical and Elastic Behavior of PBF-Block-PEO Copolymers

The mechanical and elastic performance of the copolymers was investigated in the uniaxial tensile tests under the static and cyclic deformation as well as the Shore hardness measurements. The mechanical parameters together with materials’ densities are collected in Table 3.

**Figure 8:**
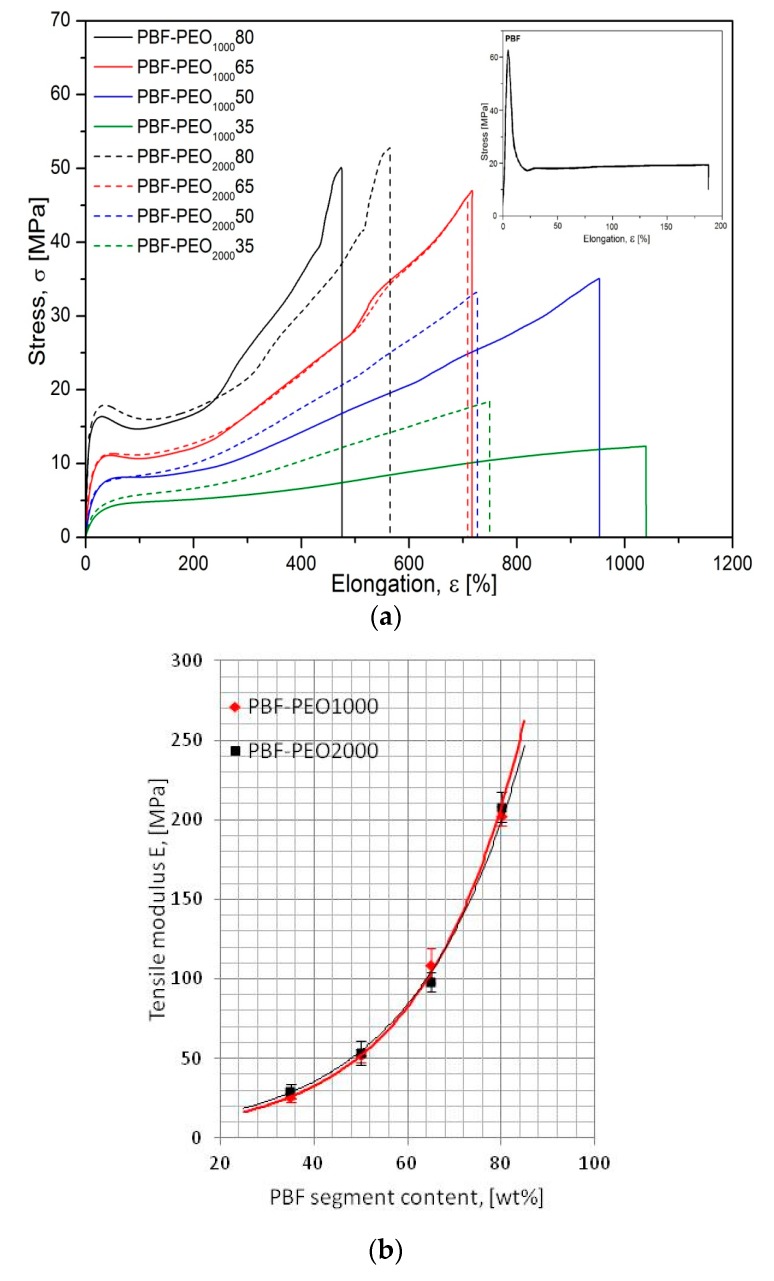
The uniaxial tensile tests’ characteristics of PBF homopolymer (inset picture) and PBF-block-PEO copolymers (**a**) and the E modulus vs. PBF segment content relationship (**b**).

As expected, the stress–strain characteristics, presented in Figure 8a, reveal significant changes in the mechanical behavior of the copolymers, particularly in deformation ability and stiffness, when compared to the neat PBF. It is due to the improved flexibility of the macromolecules by much more elastic PEO segments’ distribution, but also a specific microstructure formed. Such effects have been already observed for the PBF block copolymers with poly(tetramethylene glycol) [15] or dimerized fatty acids [17], and the mechanical parameters, namely, the tensile strength and elongation at break of the PBF-PEO_1000_ samples are comparable to the results reported in [19]. The character of deformation is also changing with the PEO segment content in both series of the copolymers, whereas the effect of the flexible segment length is mainly pronounced by the differences in elongation at break values. In general, the copolymers are characterized by distinctly reduced tensile modulus values, even for the materials containing only 20 wt % of the flexible segment (~208 MPa vs. 1460 MPa for neat PBF). However, it is in accordance with their also relatively low hardness, which is attributed to the nature of the investigated materials. Note that the E modulus values of both series show a very good fit to the exponential curve, which would be useful for estimating the E values, also for the other copolymer compositions (Figure 8b). The copolymers with a predominance of the PBF segments demonstrate a typical for semicrystalline polymers yield effect at ~30% of elongation, subsequent necking effects and a large strengthening effect due to the strain-induced macromolecule’s orientation. At these contents of the PEO segments, the effect of their molecular weight is not obvious, because while the PBF-PEO_1000_65 and PBF-PEO_2000_65 samples behave in exactly the same way, for copolymers containing only 20 wt % of the flexible segment an improved elongation (about 100%) is observed for that containing the PEO2000. It seems to be related to an incorporation of longer and more flexible sequences of polyether between the rigid PBF blocks, which in the hard phase dominated microstructure may form larger domains of the soft phase and facilitate macromolecules’ mobility under stress. This is also confirmed by lower E modulus value of the PBF-PEO_2000_80 sample. With increasing content of the PEO segments the stress–strain characteristics are gaining an elastomeric character, where a gradual increase of elongation is accompanied by a relatively small but continuous increase of stress. The 50 and 65 wt % of PEO containing copolymers reveal a relatively low stress and hardness level, but a significant ability to deformation. In these cases, an improved elongation at break is observed for PEO1000 copolymerized materials. The clue lies in the differences in copolymers’ microstructure, induced by the crystallization ability of the flexible segment with higher length, as examined previously by the DSC and WAXS. Indeed, the PBF-PEO_2000_ copolymers reveal improved crystallinity, which means that even though in their microstructure the PEO-rich soft phase with a significantly lower glass transition temperature dominates. Thus, under testing temperature the materials are in elastic state. In fact, the crystalline nanodomains of the PBF hard phase, together with the secondary PEO-assigned crystals, may constrain the continuous amorphous phase. Furthermore, the soft phase is depleted of the PEO segments involved in secondary crystals’ forming. As a consequence, the copolymers gain in stress transfer ability, which is observed on the characteristics by more pronounced strengthening effect during the deformation, but their ability to elongation is reduced when compared to the PEO1000 containing copolymers. These findings lead to conclusion that the phase separation improved by increasing the crystalline phase content, or more precisely by partial crystallization of the flexible segment, is not favorable for copolymers’ deformability. On the other hand, the stress–strain curves for the PBF-PEO_2000_50 and PBF-PEO_2000_35 samples retain their elastomeric character. Therefore, a deeper insight into elastic deformability and reversibility of the copolymers seems to be useful in evaluating the effect of the segment length on the materials elastic performance.

**Figure 9:**
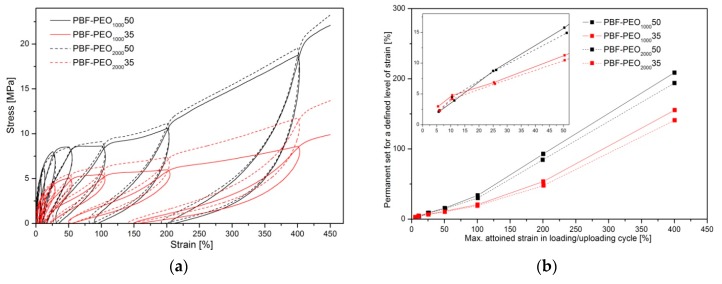
The stress–strain characteristics of cyclic tensile test (**a**) and permanent set level after following deformation cycle (**b**) for PBF-b-PEO200050 and PBF-b-PEO200035 copolymers. The zoom-in on the permanent set values in the initial deformation cycles was added as an inset picture.

For this reason, the samples were subjected to the cyclic loading/unloading tests under tension mode, and the elastic recovery after every cycle of a defined strain level was evaluated by determining a permanent set value. The smaller permanent set the better elastic recovery or higher elasticity of the material in other words. Both the stress–strain characteristics of cyclic tensile tests as well as the level of the permanent set for 5, 10, 25, 50, 100, 200, and 400% of the attained strain in the following cycle are presented in Figure 9. In general, the presented characteristics of all investigated materials reflect typical behavior of block copolymers with the features of thermoplastic elastomers: well detectable hysteresis–like curves (loops) with constantly increasing stress, and permanent set level after every loading/unloading cycle (Figure 9a). The contour made by the loops for a single material strongly corresponds to the characteristics obtained under the static tensile tests with comparable level of stress. As mentioned, the permanent set values increase with every cycle of deformation, which is particularly visible when the maximum attained strain exceeds 100%. The differences in the copolymers’ behavior resulting from the PEO segment content are getting more pronounced, and it is obvious that materials containing more rigid segments reveal higher values of the residual strain (Figure 9b). However, for the assessment of the materials’ elasticity, the most meaningful are the initial cycles of deformation. Thus, up to 10% of max. attained strain, all copolymers reveal comparable and relatively high ability to elastic recovery (permanent set values less than 5%), and it can be said that at this level of deformation, the effect of the PEO segment content and length on the copolymers’ elastic behavior is minimal. With the increase of the attained strain to 25%, the differences between copolymers containing 50 and 65 wt % of the PEO are becoming noticeable, but the effects resulting from the PEO molecular weight can be distinguished too. The elastic recovery is still relatively high with the permanent set values less than 10% for both series, however the copolymers with PEO2000 reveal slightly better elasticity, and this trend is observed in subsequent loading/unloading cycles up to the sample break. These findings are consistent with the theory about the thermoplastic elastomers, in which semicrystalline nanodomains of the hard phase, formed due to the induced phase separation, act as the physical crosslinks (knots), similar to the chemical cross-linking in the rubber. Under the tensile stress, when the continuous soft phase undergoes the deformation, the crystalline knots prevent conformational changes of the macromolecules, and ensure their return to the initial state when the force is released. Formation of the secondary crystals due to the cold crystallization of the PEO2000 segment leads to increase of the number of such nanodomains, dispersed in the continuous phase, which, in fact, “keep” the microstructure. In the PEO1000 containing copolymers, in which the effect of the phase separation is poor, the macromolecules are more liable to orientation and flow under the force, which results in larger permanent deformation observed. It is evident that the better developed phase separation in the PBF-*block-*PEO copolymers reduces the ability of samples to large elongations, but is beneficial if the elastic behavior of the materials is required.

## 4. Conclusions

The idea of this paper was to extend knowledge on the biobased PBF-*block*-PEO copolymers performance by studying the effect of the PEO flexible segment’s molecular weight (1000 and 2000 g/mol) on the resulting microstructure and mechanical performance, including elasticity of the materials. Copolymers with the PEO segments of 1000 g/mol have been already characterized by other authors, but their results are incoherent, and the materials were prepared in a glass reactor on a small scale with a very small material output. Copolymers investigated in this paper were successfully synthesized via the melt polycondensation in quantities allowing for their processing by the injection molding, and all studies were performed on the final bulk samples, reflecting the macroscopic properties of the copolymers. The expected chemical composition and a multiblocked structure of the both series of copolymers was achieved. All materials were characterized by a heterogeneous microstructure; however, the state of the phase separation is strongly dependent on the PEO segment length and content. It is becoming clear when the two series of materials are compared in details. On one hand, the PEO2000 segment supports the crystallization of the PBF segments, on the other hand for contents of at least 50 wt % and more, it is also getting crystallizable, which is evidenced by the appearance of second crystallization and melting effects in the low temperature region as well as the additional crystalline reflections on XRD diffractograms. As a consequence, the materials are characterized by the variety of resulting phase separated structures, which affect the transition temperatures of the PBF-*block*-PEO copolymers as well as their mechanical performance and elastic properties. In general, the materials reveal the characteristics typical for semicrystalline or elastomeric materials, are relatively soft and light, with ability for the high elongations (particularly PBF-PEO_1000_ copolymers), and good elastic recovery (particularly PBF-PEO_2000_ copolymers). Furthermore the materials can be easily formed by injection molding process, without shrinkage effect, which makes them suitable for the thermally molded precise elements.

## Figures and Tables

**Table 1:** The chemical composition of PBF-*block*-PEO copolymers based on ^1^H-NMR analysis.

Sample	In Feed		^1^H-NMR						
	DPx	W_PEO_	Integral Intensities				Real W_PEO_	Real W_PEO_	[η]
	mol	wt %	*I_d_*	*I_b_*	*I_e_*	*I_f_*	wt %	mol %	dl/g
PBF-PEO_1000_80	21.33	20	1.25	1.00	0.08	0.07	20.75	13.63	0.97
PBF-PEO_1000_65	9.90	35	2.50	1.00	0.10	0.11	34.38	23.98	1.11
PBF-PEO_1000_50	5.33	50	4.10	1.00	0.23	0.20	46.21	34.10	1.13
PBF-PEO_1000_35	2.87	65	7.80	1.00	0.16	0.13	62.04	49.61	1.27
PBF-PEO_2000_80	40.38	20	1.12	1.00	0.04	0.03	19.01	6.60	1.03
PBF-PEO_2000_65	18.75	35	2.69	1.00	0.08	0.07	36.05	14.51	1.07
PBF-PEO_2000_50	10.10	50	4.54	1.00	0.14	0.11	48.75	22.27	1.14
PBF-PEO_2000_35	5.44	65	8.13	1.00	0.24	0.19	63.01	33.91	1.25

DPx: degree of polymerization of PBF segment, W_PEO_: weight content of PEO unit in copolymer, [η]: intrinsic viscosity.

**Table 2:** Thermal and structural properties of PBF and PBF-block-PEO copolymers.

Sample	*T*_g_ [°C]	Δ*C*_p_ [J/g °C]	*T*_Cc_ [°C]	Δ*H*_Cc_ [J/g]	*T*_m1_ [°C]	Δ*H*_m1_ [J/g]	*T*_m2_ [°C]	Δ*H*_m2_ [J/g]	*T*c_1_ [°C]	*T*c_2_ [°C]	*x*_c_^WAXS^ [%]	*T* ^Air^_dec_	
												*T*_dec 5%_ [°C]	*T*_dec max_ [°C]
PBF	43	0.19	94	1,8	-	-	170	39	-	104	55	348	384
PBF-PEG_1000_80	4	0.47	62	28	-	-	163	35	-	-	50	354	399
PBF-PEG_1000_65	−13	0.54	44	22	-	-	154	29	-	-	46	351	394
PBF-PEG_1000_50	−25	0.65	37	18	-	-	141	20	-	-	38	342	403
PBF-PEG_1000_35	−34	0.84	45	13	-	-	118	14	-	-	33	329	405
PBF-PEG_2000_80	1	0.39	58	23	-		168	36	-	78	50	354	394
PBF-PEG_2000_65	−17	0.49	35	12	-		162	30	-	65	45	351	400
PBF-PEG_2000_50	−48	0.35	−20	7	20	12	151	20	-	96	41	344	402
PBF-PEG_2000_35	−42	0.29	-	-	28	39	136	12	−17	67	38	343	399

*T*_g1_-glass transition temperature; Δ*C*_p_: heat capacity at *T*_g_; *T*_cc_: cold crystallization temperature; Δ*H*_cc_: enthalpy of cold crystallization; *T*_m_: melting temperatures; Δ*H*_m_: enthalpy of polymer melting; *T*_c_: crystallization temperatures; *x*_c_: degree of crystallinity; *T*_dec(5%)_: polymer decomposition temperature for 5% of weight loss; *T*_dec(max)_: decomposition temperature at the highest rate.

**Table 3:** Mechanical parameters of PBF-block-PEO copolymers.

Sample	*R*_m_ [MPa]	ε_b_ [%]	*E* [MPa]	σ_y_ [MPa]	*H* [ShD]	*ρ* [g/cm^3^]
PBF	61.8 ± 2.7	255 ± 81	1460 ± 99	61.8 ± 2.7	72 ± 1.0	1.37 ± 0.005
PBF-PEO_1000_80	50.9 ± 1.4	433 ± 33	202 ± 6	16 ± 0.7	51 ± 1.3	1.31 ± 0.006
PBF-PEO_1000_65	46.1 ± 1.0	606 ± 82	108 ± 10	11 ± 0.5	46 ± 0.6	1.28 ± 0.006
PBF-PEO_1000_50	31.5 ± 2.5	924 ± 90	52 ± 5	-	38 ± 0.8	1.26 ± 0.005
PBF-PEO_1000_35	12.2 ± 0.5	1035 ± 109	25 ± 3	-	25 ± 1.2	1.22 ± 0.012
PBF-PEO_2000_80	54.8 ± 2.1	534 ± 78	208 ± 9	18 ± 0.7	54 ± 0.9	1.33 ± 0.010
PBF-PEO_2000_65	44.9 ± 1.9	641 ± 38	98 ± 5	11 ± 0.4	45 ± 0.9	1.28 ± 0.006
PBF-PEO_2000_50	30.4 ± 3.3	682 ± 66	54 ± 7	-	37 ± 1.0	1.25 ± 0.008
PBF-PEO_2000_35	16.1 ± 2.0	757 ± 63	29 ± 5	-	28 ± 1.3	1.21 ± 0.012

*R*_m_–tensile strength, ε_b_–elongation at break, *E*–tensile modulus, *H*–Shore hardness, scale D, *ρ*–density at 23 °C; the values are the average from at least six tests and the standard deviation.
